# Novel RvD6 stereoisomer induces corneal nerve regeneration and wound healing post-injury by modulating trigeminal transcriptomic signature

**DOI:** 10.1038/s41598-020-61390-8

**Published:** 2020-03-12

**Authors:** Thang L. Pham, Azucena H. Kakazu, Jiucheng He, Bokkyoo Jun, Nicolas G. Bazan, Haydee E. P. Bazan

**Affiliations:** Neuroscience Center of Excellence, School of Medicine, Louisiana State University Health New Orleans, New Orleans, LA 70112 USA

**Keywords:** Molecular biology, Neuroscience

## Abstract

The high-density corneal innervation plays a pivotal role in sustaining the integrity of the ocular surface. We have previously demonstrated that pigment epithelium-derived factor (PEDF) plus docosahexaenoic acid (DHA) promotes corneal nerve regeneration; here, we report the mechanism involved and the discovery of a stereospecific Resolvin D6-isomer (RvD6si) that drives the process. RvD6si promotes corneal wound healing and functional recovery by restoring corneal innervation after injury. RvD6si applied to the eye surface elicits a specific transcriptome signature in the trigeminal ganglion (TG) that includes *Rictor*, the rapamycin-insensitive complex-2 of mTOR (mTORC2), and genes involved in axon growth, whereas genes related to neuropathic pain are decreased. As a result, attenuation of ocular neuropathic pain and dry eye will take place. Thus, RvD6si opens up new therapeutic avenues for pathologies that affect corneal innervation.

## Introduction

Dry eye perturbs vision mainly during aging. It also occurs in rheumatoid arthritis, diabetes, thyroid gland pathologies, environmental conditions (e.g., exposure to smoke or pollutants), long-term use of contact lenses, and after refractive surgery. This pathology is triggered by a shortage in tears that lubricate, arrest infections, and nourish and sustain a clear eye surface. Corneal innervation is required to maintain the integrity of the ocular surface^1^, and nerve damage decreases tear production, blinking reflex, and perturbs epithelial wound healing, resulting in loss of transparency and vision^2–5^. For this reason, there is a strong relationship between dry eye and corneal nerve damage.

Axons from sensory nerves from the ophthalmic branch of the trigeminal ganglion (TG) neurons penetrate the corneal stroma surrounding the limbal area and branch out as the subepithelial plexus before reaching the corneal epithelium, finalizing as free nerve endings^6–8^.

After nerve damage occurs from refractive surgeries (e.g., situ keratomileusis/LASIK or photorefractive keratectomy/PRK), it takes between 3–15 years to recover corneal nerve integrity^9–11^. As a consequence, corneal sensitivity decreases, and dry-eye disease can develop, causing neuropathic pain, corneal ulcers, and in severe cases, the necessity for corneal transplants^12–14^. In addition, dry eye is linked to cold receptor function, mainly the transient receptor potential melastatin 8 (TRPM8) channels^15^ that control the corneal surface rate of cooling and maintain normal tear secretion^16–18^. In fact, a decrease in TRPM8 terminals takes place, even long after experimental corneal surgery, suggesting that these changes contribute to post-surgery neuropathic pain^19^.

Topical treatment of the neurotrophin pigment epithelium-derived factor (PEDF) plus the ω−3 fatty acid family member docosahexaenoic acid (DHA) enhances nerve regeneration and stimulates nerve regrowth in rabbit and mouse corneas after experimental surgery, as well as in pathologies like diabetes and herpes virus simplex (HSV1) infection^20–24^. Moreover, PEDF activates the Ca^2+^-independent phospholipase A2 (iPLA2ζ) activity of the PEDF receptor (PEDF-R) and releases DHA from membrane phospholipids that can be converted into bioactive docosanoids^25^, including neuroprotectin D1 (NPD1) that induces corneal nerve regeneration in a rabbit model of refractive surgery^20^. Herein, we report the discovery of a novel lipid mediator that is part of the signaling mechanism exerted by PEDF + DHA on the ocular surface. Furthermore, we uncovered that the TG genes sense corneal injury and respond to corneal stereospecific Resolvin D6-isomer (RvD6si) treatment with a specific transcriptomic signature. We demonstrate that the topical application of RvD6si is cornea protective, revealing novel mechanisms and potential therapeutic avenues for dry eye and ocular neuropathic pain.

## Results

### Identification of novel RvD6si in mouse tears

**Figure 1:**
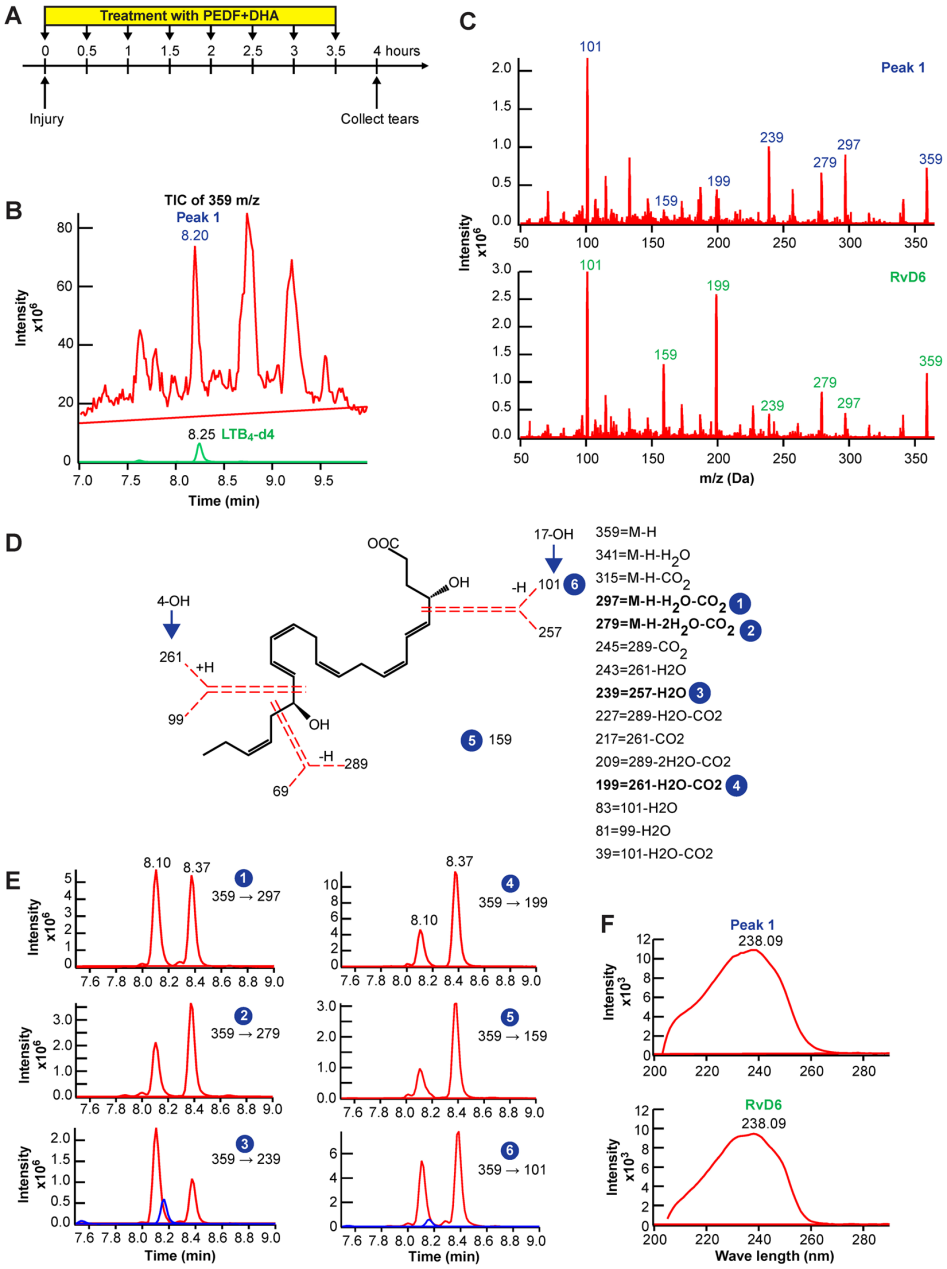
Identification of Peak 1 as a novel RvD6si from mouse tears treated with PEDF + DHA. **(A)** Experimental design with timeframe for injury, treatment, and tear samples collected from 16 corneas. **(B)** The total ion current (TIC) analysis of 359 m/z compounds (red) in the sample at the RT from 7 to 9.5 min. There are 3 peaks detected with the m/z of 359, which are regarded as dihydroxy-DHA products. In this study, we focus on the peak with an RT of 8.20 min (Peak 1). The LTB4-d4 internal standard (green) eluted at 8.25 min. **(C)** Full fragmentation analysis of selected Peak 1 and RvD6 standard. **(D)** Structural interpretation of Peak 1 with the mass of fragmented products after the collision (the dotted red lines represent the broken bonds). The fragments numbered from 1 to 6 were used for the MRM detection. **(E)** Co-injection of Peak 1 and RvD6. In this run, Peak 1 eluted at 8.10 min while the RvD6 eluted at 8.37 min (the blue color LTB4-d4 internal standard eluted at 8.15 min). All product ions matched with the same difference of RT. **(F)** The UV diode array profiles for Peak 1 and RvD6 with maximal absorbance at 238.09 nm.

The biological activities of PEDF + DHA have been revealed previously by our laboratory^20–24^. A mechanistic link of PEDF + DHA action on corneal nerve regeneration has been uncovered with the activation of the iPLA2ζ and the increased expression of the neurotrophic factors, brain-derived growth factor (BDNF) and nerve growth factor (NGF), and the axon growth guidance semaphorin 7a (Sema7A) released in tears^25^. To define which docosanoids are produced after the release of DHA by PEDF activation, mouse corneas were injured and treated, tears collected, and lipids extracted and analyzed by LC-MS/MS (Fig. 1A). The total ion chromatogram (TIC) of 359 m/z represented all di-hydroxy DHA isomers in tears after 4 h of treatment, and three peaks were well defined with retention times (RT) 8.20, 8.74, and 9.20 min (Fig. 1B). The internal standard LTB_4_-d4 (green) eluted at 8.25 min. We focused on the peak eluted at 8.20 min (Peak 1) that displays a parent ion 359 m/z upon full fragmentation, showing at least 6 matched product ions (daughter ions) compared to the RvD6 standard (Fig. 1C) with two hydroxy-groups at Carbon number 4 and 17 (Fig. 1D). When Peak 1 was co-injected with RvD6 at the same concentration, Peak 1 eluted 0.27 min earlier than RvD6 at 6 major multiple reaction monitoring (MRM) channels 359 −> 297, 279, 239, 199, 159, and 101 (Fig. 1E). The UV spectra for Peak 1 and RvD6 showed maxima absorbance (λ_max_) at 238 nm, revealing that both compounds have a conjugated diene structure (Fig. 1F). When taken together, our data support that Peak 1 is an RvD6si that shares a full fragmentation pattern with RvD6, at least 6 matched daughter ions, 2 hydroxy-groups at C4 and C17 of the DHA backbone, and UV spectrum but has different RT.

### RvD6si is derived from DHA

**Figure 2:**
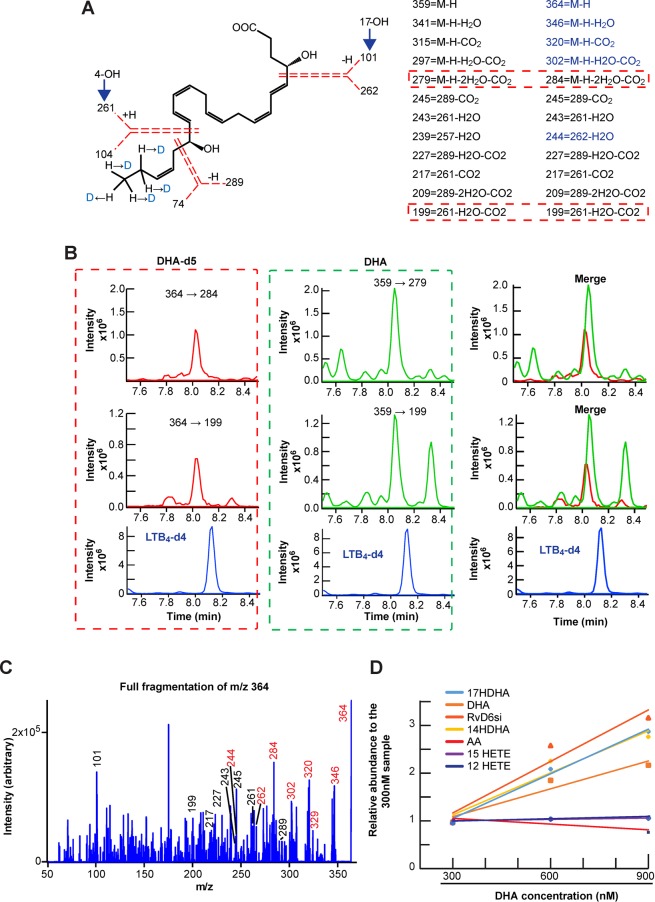
RvD6si derived from added DHA. **(A)** Structure of RvD6si-d5 with the mass of fragmented products after the collision (the dotted red lines represent the broken bonds). Five deuterium (D) originated from DHA-d5 shift the m/z of RvD6 from 359 (left column) to 364 (right column). The shifted product ions contain deuterium labeling at C21 and C22 (blue color). For MRM detection, one shifted and one non-shifted-product ions were used (red dotted boxes). **(B)** The MRM detection for RvD6si derived from DHA-d5 (red dotted box) or regular DHA (green dotted box). The transition MRM detection method is shown on top of each graph. The blue color peak is LTB4-d4 internal standard. The merge window shows that RvD6si, derived from the DHA-d5 or DHA, is eluted at the same RT, meaning that they are identical compounds. **(C)** Full fragmentation analysis for the RvD6si-d5 from B. **(D)** Quantification of RvD6si at increased DHA concentrations. The free DHA and its derivatives such as 14-HDHA, 17-HDHA, and RvD6si were gradually increased as a function of DHA concentration while free AA and its derivatives 12-HETE and 15-HETE were not.

To investigate whether the new RvD6si originated from the added DHA, an *ex vivo* corneal organ culture model was employed (16 corneas/sample). The injured corneas were cultured for 4 h in the presence of DHA or deuterium-labeled DHA (DHA-d5) plus PEDF, and the lipids from the media were extracted and analyzed. Since 5 atoms of deuterium (D) are attached to the end of the DHA backbone (at the 21^st^ and 22^nd^ C), the total mass of RvD6si-d5 was shifted to 365 Da (the [M-H] m/z is 364 in MS results) while some of its product ions were not changed after fragmentation (Fig. 2A). The MRM detection method was designed to capture the DHA-d5 total structure. The RvD6si-d5 was detected in the media with a similar RT to the RvD6si produced by PEDF + DHA (Fig. 2B). The full fragmentation of RvD6si-d5 confirmed the structure as well (Fig. 2C). In addition, the origin of the RvD6si was validated at three different concentrations of added DHA (Fig. 2D) with an enhanced synthesis as a function of increased DHA concentration. When analyzing possible arachidonic acid (AA)- and DHA-hydroxy derivatives (HDHA), the results showed a proportional increase of DHA products such as 14- and 17-HDHAs while the amount of AA and its hydroxyl derivatives 12- and 15-HETEs were not changed. These data strongly support that the new RvD6si originates from exogenous DHA.

### RvD6si enhances corneal wound healing and recovery of corneal sensitivity after injury

Although the 2D structure of the new RvD6si matched RvD6, the different RT could make them distinct in their biological activities. To obtain enough RvD6si for testing, 60 mice were injured and treated with PEDF + DHA every 30 min for 4 h, and the tears collected. The next day, the mice were euthanized, and the corneas isolated and incubated in media with PEDF + DHA for 4 h. The lipids extracted from tears and corneal media were combined and run in UPLC employing a C18 column, and fractions were collected every 30 sec from 6 to 12 min. All fractions were subject to lipidomic analysis to detect the availability of the new RvD6si. Fractions 6 to 8 with clear detectable amounts of RvD6si were pooled (Supplementary Fig. S1). The purity of our targeted lipid mediator was determined by lipidomic analysis before being tested *in vivo*. The isolated RvD6si showed very low contamination of other DHA derivatives as well as AA, eicosapentaenoic acid (EPA), and their derivatives (Supplementary Fig. S1).

**Figure 3:**
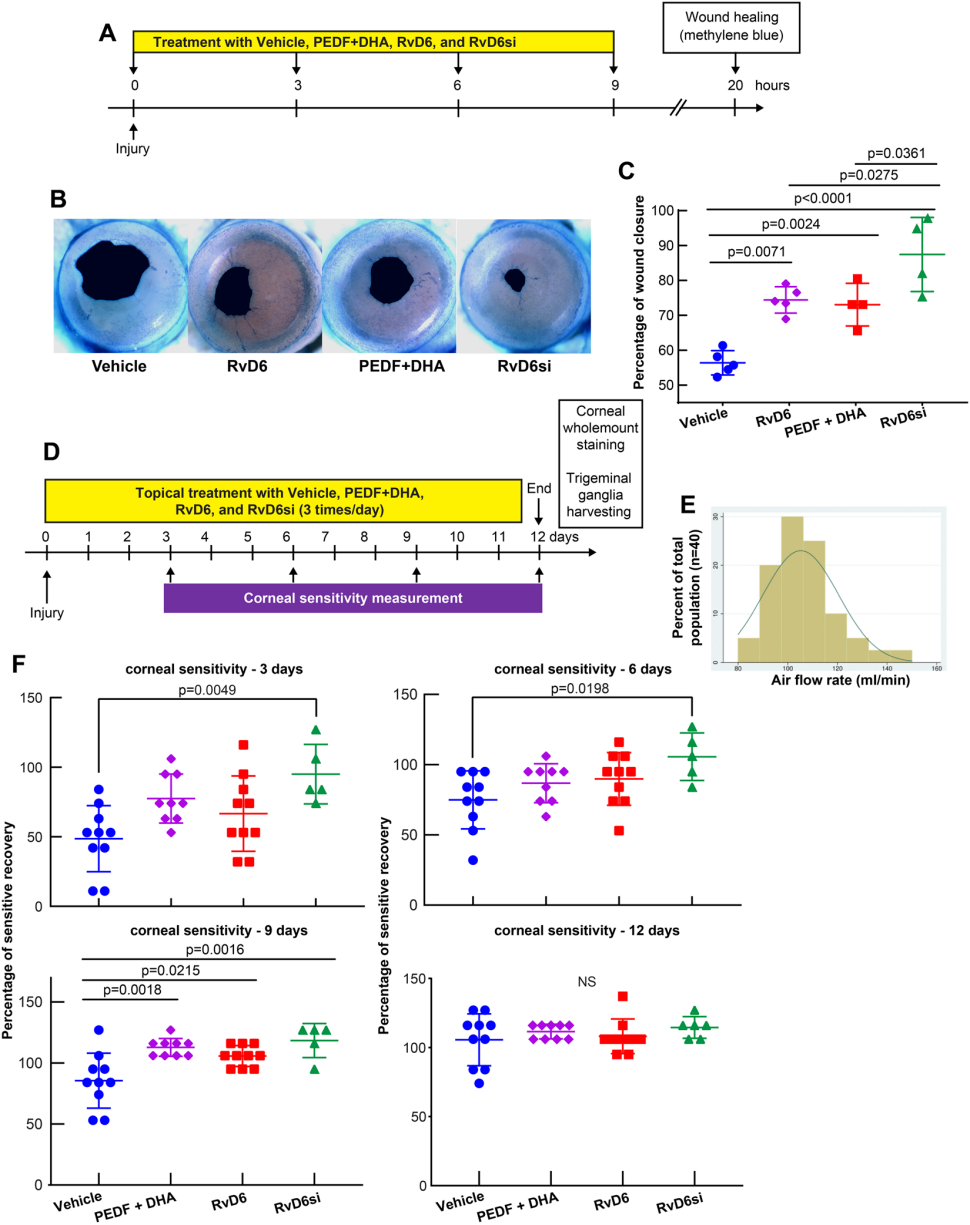
RvD6s enhance corneal wound healing and sensitivity. **(A)** Experimental design of wound healing experiments. **(B)** Representative images of corneal wounded area stained with methylene blue 20 h after injury. **(C)** The calculated wound closure after injury and treatment. **(D)** Experimental diagram of corneal sensitivity and collection of cornea and TG tissues. **(E)** Distribution of recorded corneal sensitivity in the non-injured mouse using a non-contact aesthesiometer (N = 40 corneas). **(F)** Corneal sensitivity recorded every 3 days. RvD6si-treated mice have significantly higher sensitivity at day 3, 6, and 9, while PEDF + DHA- and RvD6-treated groups showed higher cornea sensation only at day 9. At day 12, there was no difference between the tested compounds and vehicle. The statistical p-value is derived from one-way ANOVA, followed by Tukey’s honest significant difference (HSD) multiple pairwise comparisons.

Previous studies have shown that PEDF + DHA promotes corneal wound healing in rabbit^20,21^, and in normal and diabetic mice^24,25^ after experimental surgery. We tested the ability of RvD6s (either RvD6 or RvD6si) in stimulating corneal wound healing. The right mouse eyes were injured, and the animals were divided into four groups: vehicle, PEDF + DHA, RvD6, and RvD6si (Fig. 3A). Twenty h after injury, all drug-treated mice had faster corneal wound healing than vehicle; however, the greatest increase was found in the animals treated with the RvD6si (Fig. 3B,C).

Corneal sensitivity was evaluated at days 3, 6, 9, and 12 after corneal injury and treatment (Fig. 3D). A new methodology to measure the sensation in mouse cornea was introduced using the Belmonte non-contact aesthesiometer. Figure 3E shows a Gaussian-curve of distribution from basal corneal sensation recorded-values (n = 40 corneas) at a flow rate of 100.45 to 110.05 ml of air/minute (α = 0.05). It is important to note that this range of normal corneal sensitivity is critical to evaluate corneal sensation since the Belmonte non-contact aesthesiometer working flow rate is from 20 to 200 ml/min. The range of normal corneal sensitivity from 100.45 to 110.05 ml in the mouse was regarded as successful recovery after injury and treatment, and it was used to normalize the measurements. There was a faster recovery of corneal sensation in the animals treated with the RvD6si at 3 and 6 days after injury (Fig. 3F). By 9 days, the three treatments increased the sensitivity compared to vehicle, and at 12 days, there was no significant difference in any of the studied groups.

### RvD6si activates corneal nerve regeneration

**Figure 4:**
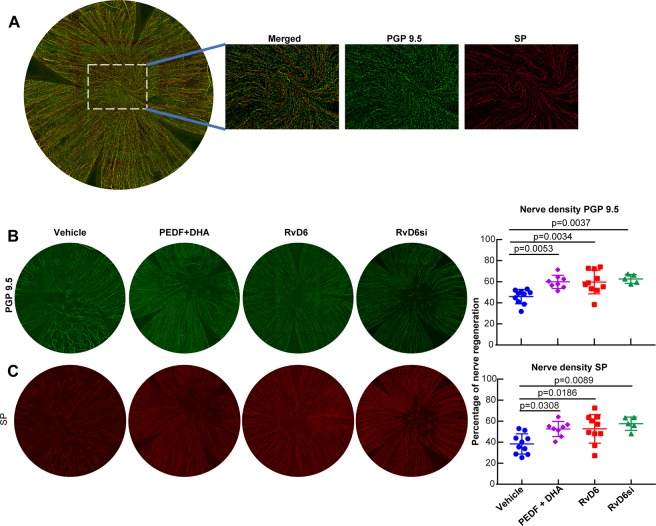
RvD6s enhance corneal nerve regeneration. **(A)** Whole-mount images of normal corneal nerves stained with anti-PGP9.5, a pan-marker for total corneal nerves and SP, a major neuropeptide in the mouse cornea. The insets, which are marked by a dashed box in the whole-mount images, show the amplified center area of the cornea with double PGP 9.5 and SP staining, and PGP 9.5 and SP alone. **(B,C)** Representative wholemount images and calculated nerve density of PGP 9.5 (**B**) and SP (**C**) positive axons at 12 days after injury and treatment. Data were normalized to the baseline (uninjured corneas in **A**). The statistical p-value is derived from one-way ANOVA, followed by Tukey’s honest significant difference (HSD) multiple pairwise comparisons.

PEDF + DHA stimulates corneal nerve regeneration in injury animal models^20–25^. It was important to confirm the biological activity of RvD6si as a lipid mediator underlying the action of PEDF + DHA. To test this hypothesis, mice were injured and treated (as described in Fig. 3D). Isolated corneas were stained with PGP 9.5, a pan-neuronal marker, and with SP neuropeptide antibodies. The density of non-injured corneal nerves positive to PGP 9.5 and SP, respectively, was used to normalize the values (Fig. 4A). Substance P is a main neuropeptide in mammalian corneas^26–28^. Moreover, a previous study from our group has demonstrated that there is a correlation between corneal sensitivity and SP-positive nerves^29^.

At 12 days after injury and treatment, total corneal nerve density was 45.9 ± 6.8% of the normal cornea in the vehicle-treated group and significantly higher in the RvD6si treated corneas 62.6 ± 4.2% (p < 0.05) (Fig. 4B). PEDF + DHA and RvD6 treatment also increased nerve density to 59.9 ± 63% and 59.7 ± 11.2%, respectively. There were no significant differences between RvD6si, RvD6, and PEDF + DHA. Similarly, the density of SP-positive nerves at 12 days after injury was higher with RvD6si, RvD6 and PEDF + DHA treatments compared to the vehicle-treated group (Fig. 4C). This result confirmed a faster recovery of corneal sensitivity in treated corneas (Fig. 3F) and strengthened the biological function of the RvD6si as the main mediator in the mechanism of PEDF + DHA to enhance corneal nerve regeneration.

### RvD6si modulates a transcriptome signature in the trigeminal ganglion

**Figure 5:**
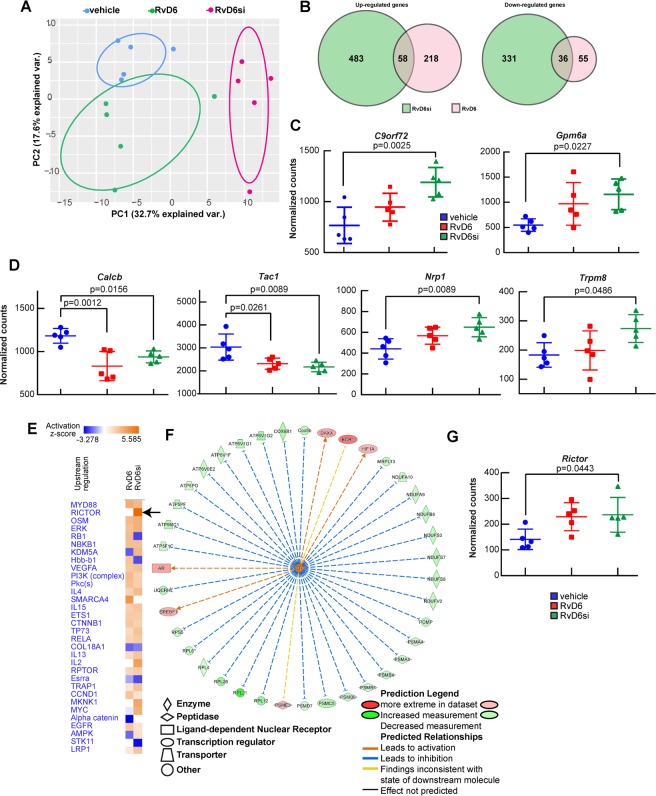
Changes in the TG transcriptome after cornea injury and RvD6s treatment. **(A)** The principal component analysis of TG RNA-sequencing data demonstrates well-clustered transcriptional profiles of the three groups of analyzed samples. **(B)** The Venn diagram of shared up- and down-regulated genes between RvD6 (pink) and RvD6si (green) with vehicle samples as reference. Inputted genes are significantly different from RvD6s compared to vehicle group (FDR < 0.05). **(C)** The box plot of two significant increase genes in the axonal growth cone classification (GO-0044295). **(D)** Changes in genes involved in inflammation and pain. (**E**–**G**) Evidence of *Rictor* gene involvement in the nerve regenerated mechanism of RvD6si. **(E)** The upstream analysis heatmap of RvD6si_vs_vehicle and RvD6_std_vs_vehicle show significant genes changes. The RICTOR is marked with bold black arrows. **(F)** The detail signaling pathways of RICTOR in RvD6si_vs_vehicle comparison is shown in the middle panel. The blunt blue arrows represent inhibited interaction, the red tip arrows represent activated interaction, and the yellow arrows represent conflicted interaction by the IPA analysis. **(G)** The box plot of *Rictor* gene expression. The statistical p-value is derived from one-way ANOVA, followed by Tukey’s honest significant difference (HSD) multiple pairwise comparisons.

Because corneal sensory nerves originate in TG neurons, we explored if corneal injury could be sensed in the TG and t, in turn, would elicit a gene expression response. Thus, TG were harvested 12 days after injury and treatment with RvD6si or RvD6 or vehicle treatment used as control (Fig. 3D), and then RNA-seq analysis was performed. Quality controls showed that mapped reads range from 84.63 to 93.00%, with about 20,000 expressed genes / sample. Principal component analysis (PCA) showed good separation of vehicle-treated from the RvD6si- or RvD6-treated groups (Fig. 5A). The two RvD6s shared 58 upregulated genes and 36 downregulated genes compared to controls (Fig. 5B). To classify upregulated genes of RvD6si_vs_vehicle and RvD6_vs_vehicle, gene enrichment analysis was used to demonstrate that the RvD6si showed a difference in cellular comparted locations (Supplementary Fig. S2) and that activate axonal growth cone genes (gene ontology number 0044295). The box plots depict two activated genes by RvD6si in this class: *C9orf72* and *Gpm6a* (Fig. 5C). We also detected specific genes related to neuropeptides and ion channel receptors in the cornea that are stimulated by the addition of PEDF + DHA^19,21,24^ (Fig. 5D). The RNA-seq established that RvD6 or RvD6si reduced gene expression of two major neuropeptides, tachykinin precursor 1 (*Tac1*) that encodes Substance P (SP) and calcitonin-related polypeptide beta (*Calcb*). It is important to note that these neuropeptides, especially *Calcb*, are major pain-induced mediators in migraine and other primary headaches^30^. In contrast, the RvD6si selectively enhanced the expression of transient receptor potential melastatin 8 (*Trpm8*) channel, and neuropilin 1 (*Nrp1*), the co-receptor for several factors including class III/IV semaphorins, certain isoforms of vascular endothelial growth factor, and transforming growth factor beta^31^.

Further analysis revealed a strong induction by RvD6si of the transcriptional factor *Rictor* (Fig. 5E) that is a part of the rapamycin-insensitive mammalian target complex-2 (mTORC2) (Fig. 5E,G). There were 39 genes modulated by RICTOR modified by RvD6si. Among those, 37 (95%) genes matched the IPA knowledge collected from published data, while only two genes, *Egr1* and *Psme3*, did not fit with the prediction (yellow arrows) (Fig. 5F). It is important to note that all genes subjected to IPA analysis are significantly different (FDR < 0.05 in the DESeq2 analysis) in comparison to the vehicle-treated group. For this reason, 95% of downstream genes matched to IPA knowledge; the *Rictor* signaling is clearly stimulated in TG by RvD6si (Fig. 5G).

## Discussion

Previous studies from our laboratory have demonstrated the potential use of PEDF + DHA for corneal wound healing and nerve regeneration in post-surgical models of rabbits and mice^20–25^. This included the observation that activation of the iPLA2ζ activity of the PEDF-R releases DHA from phospholipids, suggesting that docosanoids could be synthesized in the cornea^25^. Here, we report the finding identification and characterization of its bioactivity of a novel Resolvin D6si in tears that is derived from DHA upon activation of PEDF on its receptor. The full MS/MS fragmentation of the RvD6si matches six characteristic ions with the RvD6 as well as the UV diode array profile (Fig. 1). The biological activity revealed that it enhances corneal wound healing and sensitivity recovery, more potently than PEDF + DHA after corneal PRK-mimic surgery (Fig. 3). These results indicate that RvD6si is the main lipid mediator that contributes to the signaling mechanism of the action of PEDF + DHA. Moreover, the RvD6s and PEDF + DHA treatments show similar enhancement in corneal innervation at 12 days after injury and treatment (Fig. 5).

Resolvin D6 was described in human polymorphonuclear neutrophils^32^, skin^33^, brain^34^, cerebrospinal fluid^35^, and plasma^36^. However, this is the first report demonstrating a biological function of RvD6 and of a novel stereoisomer. The formation of potent bioactive mediators from DHA was proposed when mono-, di-, and tri-hydroxy DHA-derivatives were detected as enzyme-mediated products of oxygenated metabolites of DHA in the retina^37^. Unlike the retina, where photoreceptor membranes have high DHA content esterified at the sn-2 position of phospholipids^38^, the cornea contains more AA at that position^25,39^. For this reason, the addition of exogenous DHA is required to synthesize docosanoids rather than eicosanoids. Further, the RvD6si was not detected when corneas were treated with DHA or PEDF alone (data not shown), supporting the premise that novel RvD6si is only detected when corneas are treated with PEDF + DHA. This observation is in agreement with a previous study showing that neither RvD6 nor its stereoisomers were detected in human tear samples^40^. Since the RvD6si was found primarily in the tears or media of corneas in organ culture, this suggests that the RvD6si needs to be secreted into the extracellular compartment to become functional. The biological activity may be elicited through a receptor and, in turn, modulates cell signaling and transcription factors, upregulating, as a consequence, neurotrophic genes in the cornea^25^. RvD6si may act in autocrine fashion and/or may diffuse through tears and act as a paracrine signal on other ocular surface cells.

Most of the corneal nerves originate from neurons localized in the TG^6^. Therefore, using unbiased RNA sequencing, we have deciphered here that RVD6 and RvD6si shared a small number of upregulated genes in the TG, implicating that the signaling mechanism of their biological activities have differences. The RNA-seq data reveal a strong activation by the RvD6si of two genes, *C9orf72* and *Gpm6a*, that stimulate neurogenesis and growth cone formation^41,42^. We also found genes related to pain since corneal neuropathic pain can occur after nerve damage^43^. The expression of two genes involved in pain was decreased in corneas treated with the RvD6si: *Tac1* that encodes SP, which is one of the most abundant neuropeptides expressed in corneal nerves^26–28^. SP exerts proinflammatory effects, and preclinical studies linked their action to chronic pain^44^. Besides, *Tac1* is upregulated in the TG after corneal injury supporting the finding that corneal nerves “sense” and transmit the inflammatory signal to the TG^45^. The other is *Calcb*, which encodes Calcitonin gene-related peptide (CGRP). CGRP is also abundant in corneal nerves^21^ and plays an essential role in neurogenic inflammation and pain^30^. Another important gene in this category is *Trmp8*. TRPM8 channels regulate the wetting of the ocular surface and have an analgesic effect on chronic pain^17,46–49^. Previous studies in mice where the nerves had been damaged showed that TRPM8-positive nerve fibers only reach 50% of their normal density by 3 months after the injury, suggesting that the decrease in TRPM8 nerve terminals can contribute to dry eye-like pain^19^. The increased expression of Trpm8 after injury and treatment with RvD6si suggests that the new lipid could protect corneas from pain. In addition, the selective increase of *Nrp1* is also interesting, since it is the co-receptor of SEMA3A that has been shown to attenuate mechanical allodynia in a rat model of sciatic nerve injury^50^.

Our results disclose that the RvD6si potently and selectively induces *Rictor* gene expression in the TG. As a regulator of PI3K/Akt pathways, RICTOR is a key component of mTORC2 and is clearly involved in cell proliferation and repair. In agreement with this concept, the deletion of *Rictor* or mTORC2 inhibited the sensory-axonal regeneration in mice after dorsal root ganglion injury^51^.

In conclusion, our data demonstrate that a new RvD6si produced by the injured cornea after PEDF + DHA treatment is necessary for corneal wound healing and nerve regeneration. This novel lipid mediator activates signaling that communicates from the cornea to TG neurons, and as a response, modulates specific gene signatures that enhance axon growth, decrease neuropathic pain, and foster containment of dry eye. Our findings open potential therapeutic avenues using RvD6sifor impaired-corneal nerve diseases, including dry eye, corneal neurotrophic ulcers, neurotrophic keratitis, and neuropathic pain.

## Methods

### Animals

Ten-week-old male CD1 mice were purchased from Charles River (Wilmington, MA, USA) and maintained in a 12-h dark/light cycle at 30 lux at the animal care facility at the Neuroscience Center of Excellence, Louisiana State University Health New Orleans, New Orleans, LA. The animals were handled in compliance with the guidelines of the Association for Research in Vision and Ophthalmology Statement for the Use of Animals in Ophthalmic and Vision Research, and the experimental protocols were approved by the Institutional Animal Care and Use Committee at Louisiana State University Health New Orleans.

### Corneal injury and treatment

Mice were anesthetized with a mix of ketamine (200 mg/kg) and xylazine (10 mg/kg) that was injected intraperitoneally, and one drop of proparacaine hydrochloride solution (0.5%) was applied to the right eye subjected to injury. As previously described^19,29^, the center of the cornea was demarcated with a 2 mm trephine, and the epithelium and the anterior stroma were gently removed under a surgical microscope using a corneal rust ring remover (Algerbrush II; Alger Equipment Co., Lago Vista, TX, USA). One drop of 0.3% of tobramycin ophthalmic solution (Henry Schein, Melville, NY, USA) was applied to the eye to prevent postoperative infection. The same investigator (J.H.) performed all surgeries. Afterward, 10 µl of PEDF (50 ng/ml) plus DHA (50 nM) or DHA-derived lipid mediators were applied topically, as explained in each experimental design.

### Lipidomic analysis

Five microliters of sterile PBS was instilled in the inferior cul-de-sac of the mouse eye, and 30 s later, tears were collected in 1 mL of ice-cold MeOH containing 1 g/L Butylated hydroxytoluene followed by the addition of 2 ml of CHCl_3_ and 5 μl of an internal standard mixture of deuterium-labeled lipids AA-d8 (5 ng/μl), PGD_2_-d4 (1 ng/μl), EPA-d5 (1 ng/μl), 15-HETE-d8 (1 ng/μl), and LTB_4_-d4 (1 ng/μl). The samples were sonicated in a water bath for 30 min and stored at −80 °C overnight. The next day, the samples were centrifuged, the supernatant was collected, and the pellet was washed with 1 ml of CHCl_3_/MeOH (2:1) and centrifuged, and then the supernatants were combined. Water at pH 3.5 was added to the supernatant at the ratio 1:5, vortexed, and centrifuged, and the pH of the upper phase was adjusted to 3.5–4.0 with 1 N HCl. The lower phase was collected, dried under N_2_, and then resuspended in 1 ml of MeOH and stored at −80 °C.

For corneal organ culture experiments, 2 mL of media was collected and centrifugated at 14,000 rpm for 15 min at 4 °C to remove cellular debris. Lipids were extracted by the Blight and Dyer method^52^. Briefly, 3.75 ml of a mixture of CHCl_3_: MeOH (1:2) was added to 1 ml of sample and 5 μl of the deuterium-labeled internal standard mixture of lipids. The samples were vortexed and stored at −80 °C overnight. Next, to make two phases, 2.5 ml of CHCl_3_ was added and vortexed, and then 2.5 mL of water (pH 3.5) was added, vortexed, and the pH of the upper phase adjusted to 3.5–4.0 with 1 N HCl. The lower phase was dried down under N_2_, resuspended in 1 ml of MeOH, and stored at −80 °C.

LC-MS/MS analysis was performed in a Xevo TQ equipped with Acquity I class ultra-performance liquid chromatography (UPLC) with a flow-through needle (Waters Corporation, Milford, MA). As previously described^25,53^, samples were dried under N_2_, resuspended in 20 μl of MeOH/H_2_O (2:1), and injected into a CORTECS C18 2.7 μm 4.6 × 100 mm column (Water, MA). The column temperature was set at 45 °C with a flow of 0.6 ml/min. The initial mobile phase consisted of 45% solvent A (H_2_O + 0.01% acetic acid) and 55% solvent B (MeOH + 0.01% acetic acid) and then a gradient to 15% solvent A for the first 10 min followed by a gradient to 2% solvent A for 18 min, 2% solvent A run isocratically until 25 min, and then a gradient back to 45% solvent A for re-equilibration until 30 min. Lipid standards (Cayman, Ann Arbor, MI) were used for tuning and optimization, as well as to create calibration curves for each compound. RvD6 [4S,17S-dihydroxy-5*E*,7*Z*,10*Z*,13*Z*,15*E*,19*Z*-docosahexaenoic acid] standard was a generous gift of Dr. Charles Serhan (Harvard University, Boston, MA, USA).

### Production of Resolvin D6si from mouse tears and cornea

Mouse corneas (n = 60) were injured and treated topically with PEDF + DHA for 4 h. Tears were collected in MeOH and stored at −80 °C. After 24 h, mice were euthanized, and injured corneas were excised and cultured with PEDF + DHA in DMEM/F12 media for 4 h. The medium was collected, and lipids were extracted as described above. Lipids from pooled tears and cornea-cultured media were subjected to UPLC separation using a C18 column (Water, MA). Twelve fractions (30 sec/fraction) between 6–12 min after injection were collected. The procedure was repeated at least 8 times with 25 µl of sample/run until all the sample was fractionated. Each fraction was dried under N_2_ and resuspended in 1 mL of MeOH. The presence of RvD6si in 10 µl of each fraction was confirm using the described LC-MS/MS system. The fractions with high purity and concentration of RvD6si were pooled and stored at −80 °C until needed for the *in vivo* experiments.

### Corneal wound healing

As previously described^29^, mice were euthanized 20 h after injury and treatment, and corneas were stained with 0.5% methylene blue for 20sec and then washed with PBS for 2 min. Photographs were taken with a dissecting microscope (SMZ 1500; Nikon, Tokyo, Japan) through an attached digital camera (DXM 1200; Nikon). The images corresponding to the wounded area were quantified using Photoshop CC 2014 software (Adobe, San Jose, CA, USA).

### Corneal sensitivity measurement

The non-contact corneal aesthesiometer has been described as a more reliable method than the standard Cochet-Bonnet aesthesiometer to determine the corneal sensation threshold^54^. Therefore, for corneal sensation measurement, the Belmonte non-contact corneal aesthesiometer^55^ was used with some modification. Briefly, one researcher held the mouse and kept the air output needle at a distance of 3 mm from the cornea. Another researcher controlled the air flow rate. The measurements started at an air flow rate of 80 ml per minute and then increased gradually by ten units until the mouse started blinking. When the mouse blinked, the air flow rate was recorded as the final corneal sensitivity index.

### Corneal nerve analysis

Twelve days after injury and treatment, mice were euthanized, and the eyes enucleated and fixed with Zamboni’s fixative (American Master Tech Scientific, Lodi, CA, USA) for 45 min at room temperature. As previously described^19,24,29^, the corneas were then excised and fixed for an additional 15 min, followed by 3 washes with PBS. To block nonspecific binding, corneas were incubated with 10% normal goat serum plus 0.5% Triton X-100 in PBS for 1 h at room temperature. Afterward, corneas were incubated with the primary antibodies, rabbit monoclonal anti-PGP9.5 (1:500), (ab108986; Abcam, Cambridge, MA, USA), and rat monoclonal anti-substance-P (SP; 1:100) (sc-21715; Santa Cruz Biotechnology, Dallas, TX, USA) for 24 h at room temperature with constant shaking. After being washed with PBS, the corneas were incubated with the corresponding secondary antibodies goat anti-rabbit Alexa-Fluor 488 (1:1000) and goat anti-rat Alexa-Fluor 488 (1:1000) (Thermo Fisher Scientific, Waltham, MA, USA) for 24 h at 4 °C. Four radial cuts were performed on each cornea that was flatly mounted on a slide with the endothelium side up and examined with a fluorescent microscope (Deconvolution microscope DP80; Olympus, Tokyo, Japan). The images were merged together to build the entire view of the corneal nerve network. The corneal nerve density was measured using Photoshop CC 2014 (Adobe) as previously described^26,29^.

### Trigeminal ganglion RNA sequencing

TG corresponding to the injury eye side (n = 5) were harvested and kept in RNAlater solution (Thermo Fisher Scientific) until homogenized on ice using a Dounce homogenizer. Total mRNA was extracted using an RNeasy mini kit (Qiagen, Germantown, MD, USA) as described by the manufacturer. The purity and concentration of RNA were determined with a NanoDrop ND-1000 spectrophotometer (Thermo Fisher Scientific), and the samples were stored at −80 °C until used. RNA sequencing was performed using the adapted Smart-seq2 protocol^56^. Briefly, one ng of total RNA was reverse transcribed with the Oligo-dT_30_VN and template-switching oligo (TSO) primers. The total cDNAs were amplified using ISPCR primer, and the library was made using the Nextera XT DNA library preparation kit (Illumina, San Diego, CA, USA). The libraries were pooled using the same molarity and sequenced using the NextSeq 500/550 High Output Kit v2 (75 cycles, Illumina). After demultiplexing, RNA-seq data were aligned to the GENCODE GRCm38 mouse primary genome assembly (Release M22, gencodegenes.org/mouse/) using the RSubread package v1.34.6 for R v3.6.1^57^. The outputted BAM files for sequencing data alignment were counted using featureCounts function (Subread v1.6.5 in Ubuntu LTS 16.4 operating system)^58^. Next, the raw count data were subjected to differential gene expression analysis using DESeq2 package for R^59^. The adjusted p-values were regarded as the false discover rate (FDR). Significantly changed genes (FDR < 0.05) between RvD6si_vs_vehicle and RvD6_vs_vehicle were subjected to the enrichment analysis using Enrichr^60^ and pathway analysis using the IPA (QIAGEN Inc., https://www.qiagenbioinformatics.com/products/ingenuity-pathway-analysis).

### Statistical analysis

Data are expressed as mean ± SD of ≥3 independent experiments. The data were analyzed by 1-way ANOVA followed by Tukey honest significant difference post hoc test at 95% confidence level to compare the different groups and considered significant when p < 0.05. All statistical analyses were performed using the Stata 14 (StataCorp, College Station, TX, USA). Graphs were made using Prism 7 software (GraphPad Software, La Jolla, CA, USA) and Bio Vinci (BioTuring, La Jolla, CA, USA). For the sequencing data, since the DE-Seq2 analysis does not provide the multi-samples comparison, the normalized counts from DE-Seq2 were used as the input of ANOVA test.

## Supplementary information

Supplementary Data.

## Data Availability

Completed RNA-Seq data that support the findings of this study have been deposited in Gene Expression Omnibus with the accession code GSE138685.

## References

[1] Shaheen BS, Bakir M, Jain S (2014). Corneal nerves in health and disease. Surv. Ophthalmol..

[2] He J, Bazan HEP (2012). Mapping the nerve architecture of diabetic human corneas. Ophthalmology.

[3] Hamrah P (2010). Corneal sensation and subbasal nerve alterations in patients with herpes simplex keratitis: an *in vivo* confocal microscopy study. Ophthalmology.

[4] Cruzat A (2011). Inflammation and the nervous system: the connection in the cornea in patients with infectious keratitis. Invest. Ophthalmol. Vis. Sci..

[5] He J, Bazan HEP (2013). Corneal nerve architecture in a donor with unilateral epithelial basement membrane dystrophy. Ophthalmic Res..

[6] Müller LJ, Marfurt CF, Kruse F, Tervo TMT (2003). Corneal nerves: structure, contents and function. Exp. Eye Res..

[7] He J, Bazan NG, Bazan HEP (2010). Mapping the entire human corneal nerve architecture. Exp. Eye Res..

[8] Patel DV, McGhee CNJ (2005). Mapping of the normal human corneal sub-Basal nerve plexus by *in vivo* laser scanning confocal microscopy. Invest. Ophthalmol. Vis. Sci..

[9] Erie JC, McLaren JW, Hodge DO, Bourne WM (2005). Recovery of corneal subbasal nerve density after PRK and LASIK. Am. J. Ophthalmol..

[10] Chao C, Golebiowski B, Stapleton F (2014). The role of corneal innervation in LASIK-induced neuropathic dry eye. Ocul. Surf..

[11] Kymionis GD (2009). Fifteen-year follow-up after anterior chamber phakic intraocular lens implantation in one and LASIK in the fellow eye. Semin. Ophthalmol..

[12] Linna TU (2000). Effect of myopic LASIK on corneal sensitivity and morphology of subbasal nerves. Invest. Ophthalmol. Vis. Sci..

[13] Lee BH, McLaren JW, Erie JC, Hodge DO, Bourne WM (2002). Reinnervation in the cornea after LASIK. Invest. Ophthalmol. Vis. Sci..

[14] Hovanesian JA, Shah SS, Maloney RK (2001). Symptoms of dry eye and recurrent erosion syndrome after refractive surgery. J. Cataract Refract. Surg..

[15] Rosenthal P, Borsook D (2016). Ocular neuropathic pain. Br. J. Ophthalmol..

[16] Hirata H, Meng ID (2010). Cold-Sensitive Corneal Afferents Respond to a Variety of Ocular Stimuli Central to Tear Production: Implications for Dry Eye Disease. Invest. Ophthalmol. Vis. Sci..

[17] Belmonte C, Gallar J (2011). Cold Thermoreceptors, Unexpected Players in Tear Production and Ocular Dryness Sensations. Invest. Ophthalmol. Vis. Sci..

[18] Robbins A, Kurose M, Winterson BJ, Meng ID (2012). Menthol Activation of Corneal Cool Cells Induces TRPM8-Mediated Lacrimation but Not Nociceptive Responses in Rodents. Invest. Ophthalmol. Vis. Sci..

[19] He J, Pham TL, Kakazu AH, Bazan HEP (2019). Remodeling of Substance P Sensory Nerves and Transient Receptor Potential Melastatin 8 (TRPM8) Cold Receptors After Corneal Experimental Surgery. Invest. Ophthalmol. Vis. Sci..

[20] Cortina MS, He J, Li N, Bazan NG, Bazan HEP (2010). Neuroprotectin D1 Synthesis and Corneal Nerve Regeneration after Experimental Surgery and Treatment with PEDF plus DHA. Invest. Ophthalmol. Vis. Sci..

[21] Cortina MS, He J, Li N, Bazan NG, Bazan HEP (2012). Recovery of corneal sensitivity, calcitonin gene-related peptide-positive nerves, and increased wound healing induced by pigment epithelial-derived factor plus docosahexaenoic acid after experimental surgery. Arch. Ophthalmol. Chic. Ill 1960.

[22] He J, Cortina MS, Kakazu A, Bazan HEP (2015). The PEDF Neuroprotective Domain Plus DHA Induces Corneal Nerve Regeneration After Experimental Surgery. Invest. Ophthalmol. Vis. Sci..

[23] He J (2017). PEDF plus DHA modulate inflammation and stimulate nerve regeneration after HSV-1 infection. Exp. Eye Res..

[24] He J, Pham TL, Kakazu A, Bazan HEP (2017). Recovery of Corneal Sensitivity and Increase in Nerve Density and Wound Healing in Diabetic Mice After PEDF Plus DHA Treatment. Diabetes.

[25] Pham TL (2017). Defining a mechanistic link between pigment epithelium-derived factor, docosahexaenoic acid, and corneal nerve regeneration. J. Biol. Chem..

[26] He J, Bazan HEP (2016). Neuroanatomy and Neurochemistry of Mouse Cornea. Invest. Ophthalmol. Vis. Sci..

[27] Tervo K (1982). Substance P-immunoreactive nerves in the human cornea and iris. Invest. Ophthalmol. Vis. Sci..

[28] He J, Pham TL, Bazan HEP (2019). Mapping the entire nerve architecture of the cat cornea. Vet. Ophthalmol..

[29] Pham Thang Luong, Kakazu Azucena, He Jiucheng, Bazan Haydee E. P. (2018). Mouse strains and sexual divergence in corneal innervation and nerve regeneration. The FASEB Journal.

[30] Iyengar S, Ossipov MH, Johnson KW (2017). The role of calcitonin gene–related peptide in peripheral and central pain mechanisms including migraine. Pain.

[31] Chaudhary B, Khaled YS, Ammori BJ, Elkord E (2014). Neuropilin 1: function and therapeutic potential in cancer. Cancer Immunol. Immunother. CII.

[32] Serhan CN (2002). Resolvins: a family of bioactive products of omega-3 fatty acid transformation circuits initiated by aspirin treatment that counter proinflammation signals. J. Exp. Med..

[33] Motwani, M. P. *et al*. Pro-resolving mediators promote resolution in a human skin model of UV-killed Escherichia coli-driven acute inflammation. *JCI Insight* **3**, (2018). 10.1172/jci.insight.94463 PMC5926908 29563331

[34] Marcheselli VL (2003). Novel docosanoids inhibit brain ischemia-reperfusion-mediated leukocyte infiltration and pro-inflammatory gene expression. J. Biol. Chem..

[35] Mai, N. T. *et al*. A randomised double blind placebo controlled phase 2 trial of adjunctive aspirin for tuberculous meningitis in HIV-uninfected adults. *eLife* **7**, (2018). 10.7554/eLife.33478 PMC5862527 29482717

[36] Elajami TK (2016). Specialized proresolving lipid mediators in patients with coronary artery disease and their potential for clot remodeling. FASEB J. Off. Publ. Fed. Am. Soc. Exp. Biol..

[37] Bazan NG, Birkle DL, Reddy TS (1984). Docosahexaenoic acid (22:6, n-3) is metabolized to lipoxygenase reaction products in the retina. Biochem. Biophys. Res. Commun..

[38] Anderson RE, Maude MB (1972). Lipids of ocular tissues: VIII. The effects of essential fatty acid deficiency on the phospholipids of the photoreceptor membranes of rat retina. Arch. Biochem. Biophys..

[39] Bazan HE, Bazan NG (1984). Composition of phospholipids and free fatty acids and incorporation of labeled arachidonic acid in rabbit cornea. Comparison of epithelium, stroma and endothelium. Curr. Eye Res..

[40] English JT, Norris PC, Hodges RR, Dartt DA, Serhan CN (2017). Identification and Profiling of Specialized Pro-Resolving Mediators in Human Tears by Lipid Mediator Metabolomics. Prostaglandins Leukot. Essent. Fatty Acids.

[41] Sivadasan R (2016). C9ORF72 interaction with cofilin modulates actin dynamics in motor neurons. Nat. Neurosci..

[42] Formoso K, Garcia MD, Frasch AC, Scorticati C (2016). Evidence for a role of glycoprotein M6a in dendritic spine formation and synaptogenesis. Mol. Cell. Neurosci..

[43] Goyal S, Hamrah P (2016). Understanding Neuropathic Corneal Pain–Gaps and Current Therapeutic Approaches. Semin. Ophthalmol..

[44] Zieglgänsberger W (2019). Substance P and pain chronicity. Cell Tissue Res..

[45] Ferrari G (2014). Ocular Surface Injury Induces Inflammation in the Brain: *In Vivo* and *Ex Vivo* Evidence of a Corneal–Trigeminal Axis. Invest. Ophthalmol. Vis. Sci..

[46] Parra A (2010). Ocular surface wetness is regulated by TRPM8-dependent cold thermoreceptors of the cornea. Nat. Med..

[47] Proudfoot CJ (2006). Analgesia mediated by the TRPM8 cold receptor in chronic neuropathic pain. Curr. Biol. CB.

[48] Liu B (2013). TRPM8 is the Principal Mediator of Menthol-induced Analgesia of Acute and Inflammatory Pain. Pain.

[49] Fernández-Peña C, Viana F (2013). Targeting TRPM8 for Pain Relief. *Open*. Pain J..

[50] Hayashi M (2011). Intrathecally administered Sema3A protein attenuates neuropathic pain behavior in rats with chronic constriction injury of the sciatic nerve. Neurosci. Res..

[51] Chen, W. *et al*. Rapamycin-Resistant mTOR Activity Is Required for Sensory Axon Regeneration Induced by a Conditioning Lesion. *eNeuro* **3**, (2017). 10.1523/ENEURO.0358-16.2016 PMC5234127 28101526

[52] Bligh EG, Dyer WJ (1959). A Rapid Method of Total Lipid Extraction and Purification. Can. J. Biochem. Physiol..

[53] Do KV (2019). Elovanoids counteract oligomeric β-amyloid-induced gene expression and protect photoreceptors. Proc. Natl. Acad. Sci. USA.

[54] Murphy PJ, Lawrenson JG, Patel S, Marshall J (1998). Reliability of the non-contact corneal aesthesiometer and its comparison with the Cochet-Bonnet aesthesiometer. Ophthalmic Physiol. Opt. J. Br. Coll. Ophthalmic Opt. Optom..

[55] Belmonte C, Acosta MC, Schmelz M, Gallar J (1999). Measurement of corneal sensitivity to mechanical and chemical stimulation with a CO2 esthesiometer. Invest. Ophthalmol. Vis. Sci..

[56] Picelli S (2014). Full-length RNA-seq from single cells using Smart-seq2. Nat. Protoc..

[57] Liao Y, Smyth GK, Shi W (2019). The R package Rsubread is easier, faster, cheaper and better for alignment and quantification of RNA sequencing reads. Nucleic Acids Res..

[58] Liao Y, Smyth GK, Shi W (2014). featureCounts: an efficient general purpose program for assigning sequence reads to genomic features. Bioinforma. Oxf. Engl..

[59] Love MI, Huber W, Anders S (2014). Moderated estimation of fold change and dispersion for RNA-seq data with DESeq2. Genome Biol..

[60] Kuleshov MV (2016). Enrichr: a comprehensive gene set enrichment analysis web server 2016 update. Nucleic Acids Res..

