# Supplementary material for: Novel RvD6 stereoisomer induces corneal nerve regeneration and wound healing post-injury by modulating trigeminal transcriptomic signature
Source: Sci Rep. 2020 Mar 12;10:4582. doi: 10.1038/s41598-020-61390-8 (PMC7067818; doi:10.1038/s41598-020-61390-8)
Supplement: Supplementary file 1 — Supplementary Data. [file 41598_2020_61390_MOESM1_ESM.pdf]

Supplementary Material for

Novel RvD6 stereoisomer induces corneal nerve regeneration and wound healing post-injury by modulating trigeminal transcriptomic signature

Thang L. Pham, Azucena Kakazu, Jiucheng He, Bokkyoo Jun, Nicolas G. Bazan, Haydee E. P. Bazan[1]

[1]To whom correspondence should be addressed
Email: hbazan1@lsuhsc.edu.

This PDF file includes:

Supplementary Figures S1-S2

Supplementary Figure S1

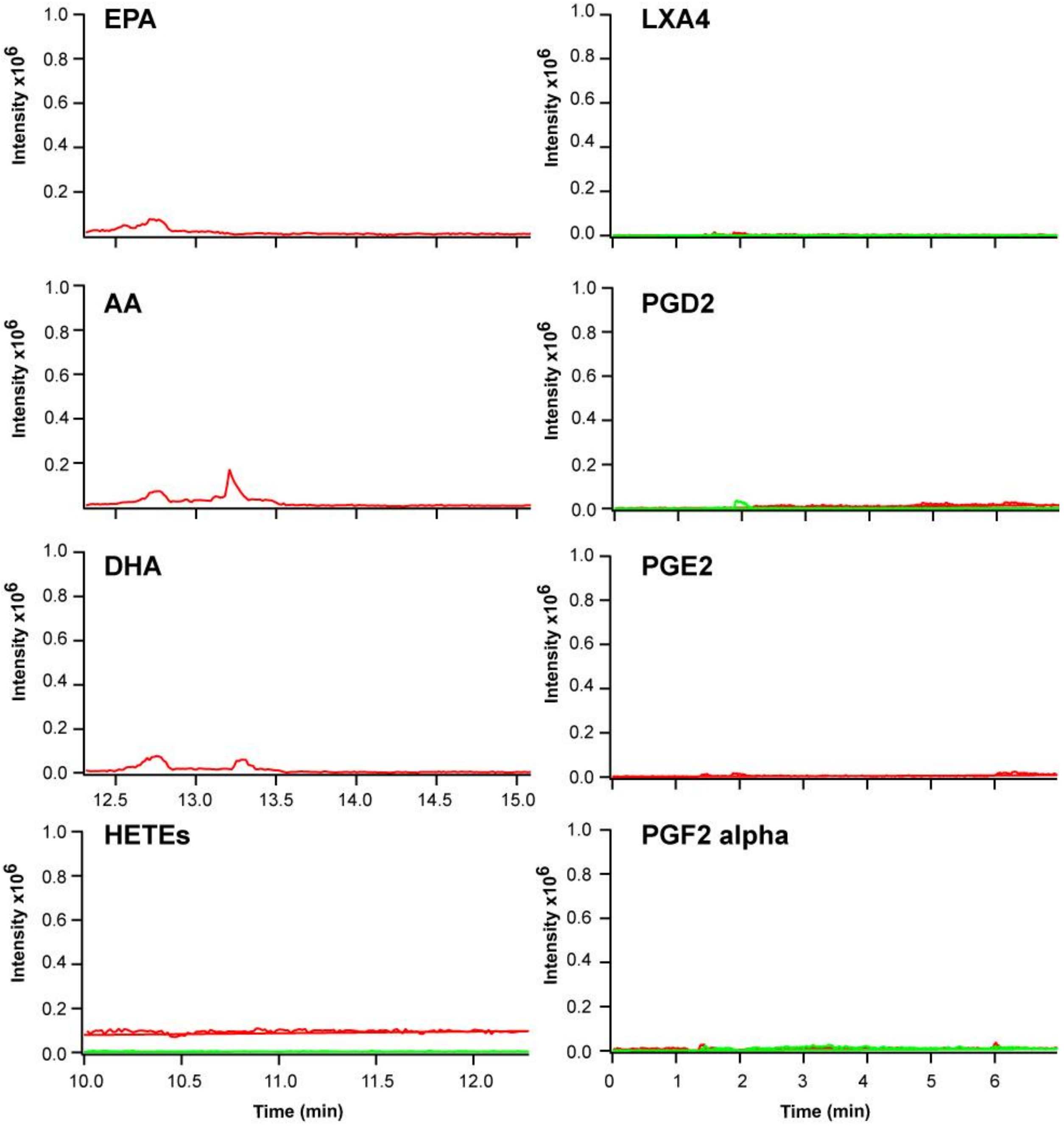

Supplementary Figure S1. High purity of isolated RvD6si from biological production. The samples from fractions 6 to 8 were pooled and analyzed using LC-MS/MS with specific MRM windows to detect DHA, EPA, and AA and its derivatives including HETEs, LXA4, PGD2, PGE2, PGF2 alpha. All MRM windows show trace amounts of targeted compounds suggesting that the isolated RvD6 is pure.

Supplementary Figure S2

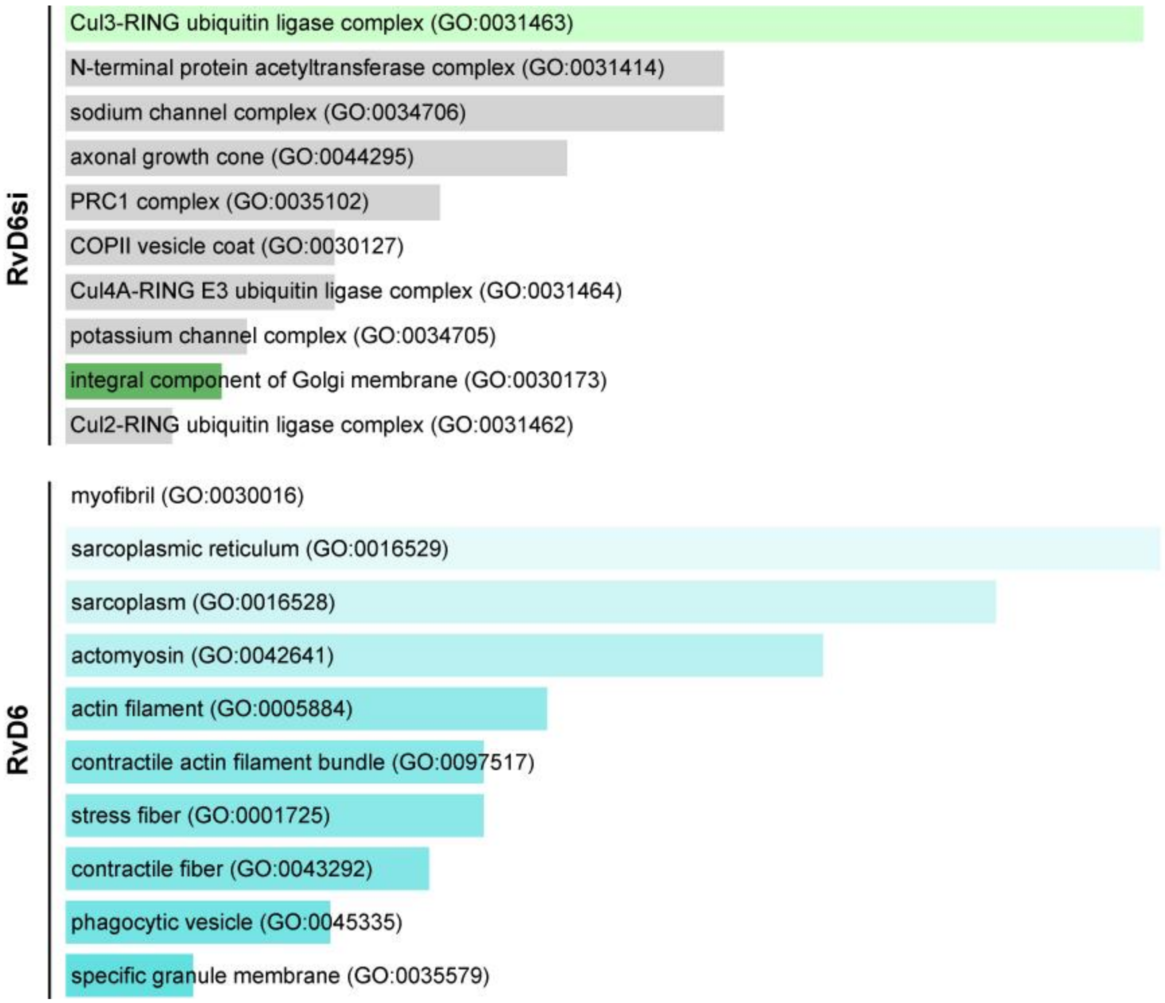

Supplementary Figure S2. The gene ontology of cellular components from Enrichr analysis. There are many groups gene located on specific cellular compartments. Among those groups, axonal growth cone (GO: 0044295) group was targeted.
